# Supplementary material for: Fitting a collider in a quantum computer: tackling the challenges of quantum machine learning for big datasets
Source: Front Artif Intell. 2023 Dec 15;6:1268852. doi: 10.3389/frai.2023.1268852 (PMC10755015; doi:10.3389/frai.2023.1268852)
Supplement: Supplementary file 1 [file Presentation_1.pdf]

Appendix to “Fitting a Collider in a Quantum Computer: Tackling the Challenges of Quantum Machine Learning for Big Datasets‘”

Miguel Caçador Peixoto, Nuno Filipe Castro,
Miguel Crispim Romão, Maria Gabriela Oliveira and Inês Ochoa

In this appendix we show the results obtained for the *Adam*-trained VQCs, namely the ROC curve for the best HP set for the QML model, shown in Figure 1. This result can be compared to the *TPE*-trained VQC results shown in the Figure 5 of the paper. As noted in the subsection 8.1 of the paper, the two optimizers are compatible.

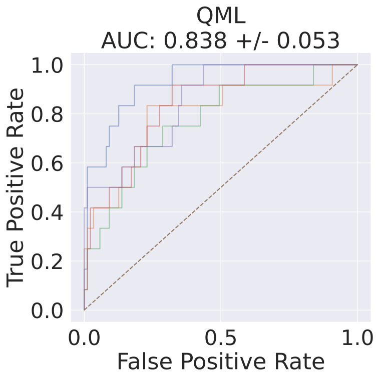

Figure 1: ROC of the best HP set, using *Adam*’s QML model average AUC score as a metric. The HP for this run are SBS for feature method, 100 data points, 1 feature, and 1 VQC layer. The corresponding shallow methods ROCs for the same data have an AUC of 0.795 ± 0.105 for SVM and 0.838 ± 0.053 for LR.

In Figure 2 we show the results obtained, using the simulation of a quantum circuit considering noise, with a larger number of random samplings and more data points. The *Qiskit.Aer* simulator for the *IBM nairobi* system was used. The corresponding noise model was assumed for the simulations and the used parameters, such as the number of qubits, number of shots, and optimization level, mirrored those considered in the section 9 of the paper. The obtained results are compatible with those shown in the Figure 5 of the paper.

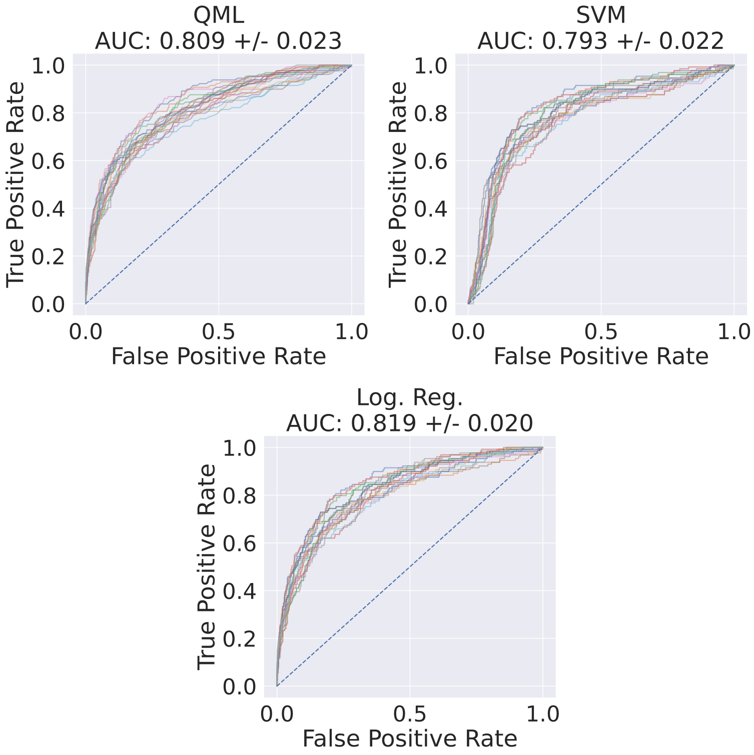

Figure 2: ROC of the best HP set, using *TPE*'s QML model average AUC score as a metric and the corresponding shallow methods ROCs. The HP for this run are SBS for feature method. 1000 data points were used. The quantum circuit was simulated considering noise. The different colours indicate each of the 20 random samplings of the data.
